# Hand-Washing Habits in a Sample of Spanish Soft Contact Lens Wearers

**DOI:** 10.3390/healthcare12212111

**Published:** 2024-10-23

**Authors:** Silvia Alonso, Irene Navarro, Genis Cardona

**Affiliations:** 1 Terrassa School of Optics and Optometry (FOOT), Universitat Politècnica de Catalunya, Violinista Vellsolà 37, 08222 Terrassa, Spain; silvia.alonso@upc.edu (S.A.); irene.navarro.giron@estudiantat.upc.edu (I.N.); 2 Applied Optics and Image Processing Group (GOAPI), Department of Optics and Optometry, Universitat Politècnica de Catalunya, Violinista Vellsolà 37, 08222 Terrassa, Spain

**Keywords:** contact lens, patient compliance, hand hygiene, microbial keratitis

## Abstract

Contact lens (CL) wear is a safe method for the correction of refractive errors. However, rare, severe ocular complications may occur which may lead to visual loss. As most of these complications are related to poor patient compliance with care and maintenance instructions, resulting in the contamination of hands, CLs and accessories, it was the purpose of this study to assess hand-washing habits in a sample of Spanish soft CL wearers. Hand hygiene was explored through a self-reported online survey and via an in-office practical demonstration in a subset of those users answering the survey. A total of 198 surveys were analyzed, and 18 CL users participated in the practical demonstration. Overall, hand-washing habits were not adequate, with 35 (17.8%) and 103 (52.2%) participants reporting not always washing their hands prior to CL or storage case manipulation, respectively. While 161 (81.3%) participants dried their hands after washing, 132 of these (82.1%) used non-disposable cloth towels. Participants receiving specific hand-washing information from their practitioners (141, or 71.1%) had better hand hygiene (*p* < 0.05). During the practical demonstration, 13 (72.2%) participants used water and soap to wash their hands, but only 3 (16.7%) displayed correct hand washing routines. Complete patient education, as well as practical reminders at all follow-up visits, are essential to ensure better hand hygiene in order to reduce the probability of ocular complications and to guarantee safe and satisfactory CL use.

## 1. Introduction

Contact lenses (CLs) are a popular option for the correction of a wide range of refractive errors including myopia, hyperopia, astigmatism and presbyopia [1]. Although CL wear is commonly considered a safe alternative to spectacles and refractive surgery, rare sight-threatening complications may occur. Amongst them, microbial keratitis (MK) has a reported annual incidence that varies with CL type and wearing modality, ranging from 1.2 to 1.9 per 10,000 wearers for daily use of rigid corneal and soft CLs, respectively, to 20 per 10,000 for overnight wear with soft (hydrogel and silicone–hydrogel) CLs [2]. A recent multicenter study from Spain estimated an annual incidence of 4 cases per 10,000 wearers, based on 304 cases diagnosed on patients mainly using monthly replacement soft CLs and multipurpose solutions [3].

Numerous studies have described a direct relationship between ocular complications and patient compliance with given instructions related to care and maintenance of CLs and accessories [4,5,6,7]. Non-compliance is a pervasive concern of CL practitioners and ocular health professionals, with published rates of non-compliance ranging from 40 to 90% [8,9]. Contact lens care obviously depends on the type and material of CL, wear modality and replacement schedule. Overall, non-compliance practices include poor hand hygiene, contact of tap water with CLs and accessories, using tap water or saline instead of CL solutions for storage, not changing the solution of the storage case each day (topping off), sleeping with or reusing daily disposable CLs, extending the replacement intervals of CLs and using solutions beyond their absolute or relative expiration dates [10,11,12,13]. Interestingly, many patients are ignorant of the critical relevance of proper care and maintenance habits or actually believe their practices are adequate to prevent complications. For instance, research by Bui and co-workers noted that, while 86% of their patients identified themselves as compliant, only 34% of these actually exhibited good levels of compliance when their practices were explored in depth [14].

Hand hygiene, in particular, is a critical aspect in healthcare settings, playing a fundamental role in preventing infections and ensuring patient safety. As such, the World Health Organization (WHO) and the Centers for Disease Control and Prevention (CDC) have established extensive guidelines and have provided user-friendly, easy to understand infographics, in which the importance of hand hygiene across all health disciplines is highlighted as a means to reduce the risk of infections [15]. However, adherence to hand hygiene protocols remains suboptimal in many healthcare settings. For instance, a meta-analysis of 105 studies exploring hand hygiene practices amongst physicians and nurses, as determined by direct observation, revealed weighted pooled compliance rates of 45% (95% CI: 40–49%) and 52% (95% CI: 47–57%), respectively [16]. Moreover, low to moderate certainty of evidence was reported in a review of 26 studies exploring several strategies based on WHO recommendations to improve hand hygiene compliance, including different types of education, oral and written reminders or performance feedback [17]. 

Although hand hygiene requirements for a health worker may not be expected of CL wearers, proper hand washing remains the most basic principle to reduce eye infection events [18,19]. Indeed, cross-contamination and pathogen tropism between hands, CLs, CL accessories, such as storage case or solution bottles, and ocular surface is evidenced by the fact that the risk of developing MK was found to be 13 times higher for patients failing to wash their hands before handling their CLs [20]. Also, the main pathogens responsible for ocular complications have been identified in storage cases, suggesting a contaminated storage case as a vector for ocular infection [21,22]. Unfortunately, non-compliance among CL wearers with hand hygiene has been reported as high as 50% in some studies [23,24,25,26,27]. For instance, in a recent survey of 950 daily disposable CL wearers, 41% of respondents did not always wash their hands with soap prior to CL insertion, 15% never washed their hands or never used soap, and 65% did not dry them with a disposable paper tissue [27]. 

Proper hand hygiene practices are critical for all CL types and wearing regimes, and particularly for non-disposable soft CL wearers, who follow a biweekly or monthly replacement schedule, or rigid CL wearers with longer replacement schedules, as this allows ample opportunity for the extensive colonization of CLs and storage cases. In effect, biofilm formation in storage cases has been observed as early as two weeks into use and is associated with inherent microbial resistance [28]. In this regard, the annually updated international survey of trends in CL prescribing documents a progressive shift towards daily disposable soft CLs (including hydrogel and silicone-hydrogel) wear over other non-disposable soft CL options, albeit with notable differences among countries [29]. Thus, for instance, while daily disposable CLs represent 70% of the whole CL market in the United Kingdom and 48% in Australia, in the United States and Spain non-disposable options are still predominant, with a market share of 63% and 60%, respectively [29]. The predominance of non-disposable CL wear in Spain was also noted in the multicenter study mentioned above, which reported a slightly superior estimated incidence of MK than previously documented in studies conducted in countries favoring daily disposable options [3]. 

Given the relevance of patient compliance and the described national particularities in soft CL prescribing, it was the aim of the present study to determine hand hygiene practices in a representative sample of soft CL users from Spain. As far as we know, no previous research has explored in detail this specific aspect of CL wear in the Spanish population. For this purpose, a two-phase study was designed: first, an online survey was distributed among Spanish soft CL wearers to gather self-reported habits regarding hand hygiene; second, hand-washing practices were assessed through in-office demonstrations in a subset of those wearers answering the survey. 

## 2. Materials and Methods

A two-phase cross-sectional observational study was designed to assess hand hygiene habits in a sample of Spanish CL wearers.

### 2.1. Phase One: Online Survey

A self-reported custom survey (available upon request from the authors) was created using Google Forms (Google LLC, Mountain View, CA, USA). The survey, conducted in Spanish, was divided into four sections and took approximately five minutes to complete. It contained a mix of mandatory and optional questions to allow respondents to bypass items irrelevant to their CL usage. The first section included a short introduction in which the main purpose of the study was described (in general terms, to prevent influencing subsequent responses). A contact email was provided for participants requiring additional information. The second section collected demographic (age, sex) and CL use (CL experience in years and replacement schedule) details. The third section explored hand hygiene habits and included questions regarding frequency of hand washing prior to CL and storage case handling, methods used for hand washing and drying and changes in hand-washing habits following the COVID-19 pandemic, amongst others. Finally, the fourth section asked participants about their relationship with their CL practitioner: whether and how the patient was informed regarding hand hygiene and whether their hand hygiene habits were assessed in a practical demonstration during the CL fitting and follow-up visits. The survey was based on the clinical experience of the authors and on published literature on hand hygiene, CL wear and compliance. Prior to distribution, the survey was tested on a sample of convenience of five CL wearers, and their comments and suggestions were considered to improve the clarity of exposition and the overall flow of the survey. 

The survey link was distributed through personal and social networks of the authors between 15 July 2023 and 10 September 2023, and again between 10 February 2024 and 15 March 2024, using a snowball sampling strategy, where participants were encouraged to invite acquaintances to join the survey. Participation was voluntary, responses were anonymous and no identifiable data were collected, although respondents were invited to share their email details. Consent was explicitly obtained by answering a yes or no question at the start of the survey. 

All soft CL users were eligible to participate in the study, irrespective of CL wear modality, replacement schedule and CL wear expertise. Given the particular care and wear requirements of other CL options, such as rigid corneal and scleral lenses or orthokeratology, these respondents were excluded from this study. This survey was part of a broader research project on CL compliance, approved by the Institutional Review Board at the Universitat Politècnica de Catalunya (protocol code 12A/2018). The study adhered to the principles of the Declaration of Helsinki.

### 2.2. Phase Two: Practical Demonstration

For the practical demonstration part of the study, a subset of participants was recruited from those respondents who provided an email address in the initial survey. All respondents providing their contact details were invited to participate in this part of the study. None of the participants received any compensation for their involvement in the study.

Participants in the practical demonstration were instructed to wear their CLs on the appointed day and to bring their accessories, including bottles of solution and storage cases. Once in the research lab, they were asked to remove their CLs, as they would do at home at night, place them in their storage case and then to insert them back into their eyes, as they would do in their morning routine. Their performance was observed by three independent examiners and all aspects of CL and accessories care were graded as either correct or incorrect by consensus. Participants were not told the purpose of the demonstration until the end, when feedback was provided on their habits and education, if necessary, was reinforced. 

Although all aspects of CL wear and care were explored in the practical demonstration, only the habits regarding hand hygiene shall be reported in this article. In particular, participants were assessed on whether they washed their hands prior to CL manipulation; on their use of sufficient soap to cover the surfaces of the hands; on their actual hand-washing routine, as compared to the WHO recommendations; and on whether they employed a cloth towel or a paper tissue to dry their hands.

### 2.3. Data Analysis

Statistical analysis was conducted using IBM SPSS Statistics v.28 (IBM Corp., Armonk, NY, USA). Survey responses were reviewed and cleaned, if necessary, and were summarized using frequencies for qualitative data and mean and standard deviation (SD), or median and range, according to normality, for continuous numerical data, with non-parametric analysis applied for inferential statistics (Mann–Whitney test for continuous data and chi-square test for nominal data). To explore the influence of age as a risk factor of hand hygiene non-compliance, three age groups were defined: equal to or younger than 30 years, between 31 and 40 years and 41 years or older. A *p*-value of 0.05 or less was considered to indicate statistical significance.

Required sample size of the initial survey was estimated with the freely available Qualtrics^XM^ Sample Size Calculator (Provo, UT, USA) considering that, according to market research, 4.74% of the Spanish population between 15 and 64 years of age uses CLs, that is, about 1,500,000 wearers. Considering a confidence interval of 95% and a margin of error of 5%, the required sample size is 385. 

## 3. Results

### 3.1. Sample Demographics

A total of 203 surveys were retrieved. Following manual review, five surveys were excluded, resulting in 198 complete surveys for analysis. Reasons for exclusion were incomplete or incongruous survey responses (3) and rigid corneal CL use (2). Participants had a median age of 32 years, with a range from 18 to 64 years, and were predominantly female (154, or 77.8%). A value of 71 (35.9%) respondents were aged 30 years or less, 74 (37.4%) between 31 and 40 years, and 53 (26.7%) were 41 years of age or older. Sex distribution amongst the three age groups was similar (*p* = 0.437). 

Most respondents had between 2 and 4 years of experience with CLs (87, or 44.4%), followed by more than 4 years (77, or 39.0%) and less than 2 years (34, or 16.6%). Regarding replacement schedules, 156 (78.8%) participants wore monthly and 42 (21.2%) daily replacement CLs. Contact lens wearing experience and replacement modality were not dependent on age (*p* = 0.645 and *p* = 0.091, respectively).

### 3.2. Survey Responses

Some of the most relevant responses to the survey are summarized in Table 1.

Main reasons for not washing hands were “forgetting to do so” (46, or 80.7% of respondents not always washing hands) and “being in a rush” (6, or 10.5%), with a small percentage of participants (5, or 8.8%) reporting “it is not necessary to do so”. No statistically significant differences were found in hand-washing habits depending on age group (*p* = 0.761), sex (*p* = 0.153), CL experience (*p* = 0.216) or replacement schedule (*p* = 0.577). Two-thirds of respondents (128, or 64.6%) reported similar hand-washing procedures to those they conducted before the COVID-19 pandemic. Age was found to influence the probability of reporting a change in hand-washing procedures because of the COVID-19 pandemic (*p* = 0.014), with older respondents noting an increased awareness of the need for proper hand hygiene. 

Regarding storage case management (monthly replacement users), more than half (86, or 55.2%) of those respondents not washing their hands before handling this accessory considered it was not necessary to do so. No statistically significant differences were found in hand washing prior to storage case handling depending on sex (*p* = 0.137) or CL experience (*p* = 0.517).

Participants reporting washing their hands prior to any CL or accessory interaction mostly did so with soap and water (185, or 94.9%), although 10 (5.1%) respondents reported using water only. Anecdotally, one respondent noted washing hands with CL maintenance solution. No statistically significant differences were found on the method of hand washing depending on age group (*p* = 0.435), sex (*p* = 0.145), CL experience (*p* = 0.642) or replacement schedule (*p* = 0.564).

The majority of respondents dried their hands after washing them (161, or 81.3%). The method of hand drying was not influenced by age group (*p* = 0.710), sex (*p* = 0.588), CL experience (*p* = 0.813) or replacement schedule (*p* = 0.151). Participants using a non-disposable cloth towel to dry their hands changed this towel once a week (88, or 66.7%), between one and two weeks (22, or 16.7%) or more than two weeks (22, or 16.7%).

Regarding hand-washing information provided by CL practitioners to the respondents, 141 (71.1%) of them reported receiving specific information on proper hand washing procedures, 52 (26.3%) did not receive any information, and 5 (2.6%) respondents bought their CLs online, that is, they did not have any communication with any health professional (interestingly, all respondents buying their CLs online were from the group aged between 31 and 40 years). Younger respondents had a higher probability of receiving specific information from their practitioners (*p* = 0.003). Better hand-washing practices prior to CL (*p* = 0.008) and accessory (*p* = 0.006) handling were reported by those users receiving specific information, with a larger percentage of these users noting they always washed their hands than in the group not receiving any information. Only 23 (11.6%) practitioners reviewed hand-washing instructions with their patients in subsequent follow-up visits and, in these occasions, none of the participants was required to perform a practical demonstration.

Information was predominantly through a practical demonstration during the CL fitting visit (91, or 64.5%), followed by verbal information only (21, or 14.9%), written information only (16, or 11.3%) or a combination of practical demonstration and written instructions (8, or 5.7%). Some practitioners recommended the visualization of online video tutorials, alone (1, or 0.7%) or as an addition to written information (3, or 2.1%) or to a practical demonstration (1, or 0.7%). None of the hand-washing habits described above were found to be influenced by the type of information provided by practitioners (all *p* > 0.05).

### 3.3. Practical Demonstration

**Figure 1:**
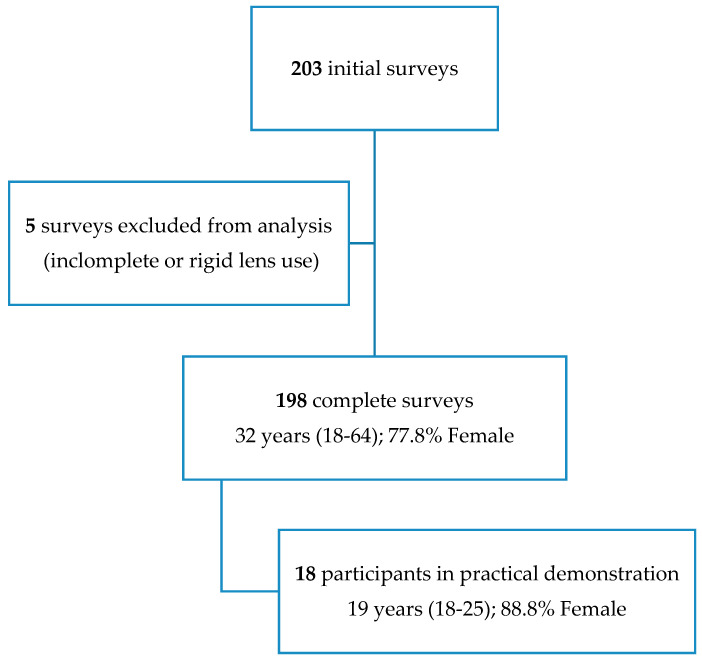
Demographics of participants in the survey and practical demonstration.

Participants in the practical demonstration (n = 18) had a median age of 19 years (range from 18 to 25 years) and 16 (88.8%) of them were female (Figure 1). Age and gender distribution in the subset of participants included in the practical demonstration was statistically different from that of the overall sample (*p* < 0.001 and *p* = 0.017, respectively). Contact lens experience of the subset of participants was similar to the overall sample (*p* = 1.000), but all of them replaced their CLs monthly. 

During the part of the practical demonstration exploring hand hygiene habits, 13 (72.2%) participants used water and soap to wash their hands and the rest (5, or 22.8%) only used water. Of those using soap, however, 9 (69.2%) failed to rub their hands as recommended by the WHO guidelines, that is, only 4 (22.2%) participants had good hand hygiene practices. After washing their hands, 10 (55.6%) and 8 (44.4%) participants used a cloth towel or a paper tissue to dry them, respectively. All considered, only three participants of 18 (16.7%) performed all hand washing routines correctly.

## 4. Discussion

The main purpose of this study was to determine the hand hygiene habits of Spanish soft CL users through an online survey and a practical demonstration. Given that monthly replacement CL schedules still predominate in Spain, increasing the chance for opportunistic ocular infection through contaminated CLs and storage cases, the most relevant finding of this research was that almost 30% of users did not wash their hands at every interaction with their CLs, a result in agreement with previous reports [23,24,25,26,27]. For instance, Morgan and co-workers documented that only about 40% of daily disposable, non-disposable and extended wear CL users displayed correct hand washing practices [24]. Similarly, Osborn and co-workers found that 41% of daily disposable CL wearers failed to wash their hands with soap before lens insertion [27]. Equally troubling was the fact that only half the respondents to the survey always washed their hands before handling their storage cases. Indeed, if the order of hand washing and accessories manipulation is not correct, the cross-contamination of these accessories may occur, which may subsequently again contaminate the hands and CLs [30]. Moreover, the hands may transfer pathogens and increase the bioburden of storage cases, which may lead to resistance to regular cleaning procedures through biofilm formation [31]. Non-compliance with hand hygiene routines was found to be generally poor irrespective of sample demographics. Indeed, age, sex and previous CL experience did not influence compliance with regard to hand washing prior to CL and storage case manipulation, in agreement with the published literature [32], although the same study reported that women were more compliant with other aspects of storage case hygiene. Interestingly, older wearers reported a change in hand hygiene practices following the COVID-19 pandemic, which was not observed in the younger groups. These findings may be in line with those described by previous researchers, who noted that while at the start of the pandemic, adolescents improved their hand hygiene practices, these good practices rapidly decreased thereafter [33]. Differences in hand hygiene routines between younger and older wearers could be related to their approach to risk management and risk awareness. Therefore, an education campaign to increase risk awareness of younger patients may have the potential to improve hand hygiene. 

Interestingly, a small percentage of users noted it was not necessary to wash their hands prior to CL manipulation, and more than half of them reported the same with reference to handling their storage cases. Upon exploring the information received from their practitioners, it was revealed that more than two-thirds of participants were provided with specific information regarding hand washing, and that this information usually consisted of a practical in-office demonstration. Although the type of information was not found to influence compliance, users receiving information of any type tended to report better hand hygiene prior to handling of CLs and accessories. These findings give support to the essential role of patient education in improving compliance, although previous researchers documented a positive initial effect of education on hand washing and a subsequent reduction over time [17,34]. Given that it has been observed that patients may forget up to 50% of the advice provided by their practitioners within minutes of leaving the office [35], and that even if handed written instructions, 63% of them never read them again at home [36], it is essential to take advantage of all follow-up visits to reinforce better practices. Indeed, multiple interventions have been found to be superior to single interactions to sustain significant behavior change [37]. Related to this, it was an unexpected finding that only 11.7% of practitioners reviewed instructions at follow-up visits, and none of them through practical demonstration. It could be recommended that, at each follow-up visit, practitioners ask their patients to perform a practical demonstration of hand washing. Subsequently, practitioners could in turn demonstrate a correct and complete hand-washing routine, emphasizing the areas where patients have shown weaknesses.

Regarding hand drying, a large percentage of participants reported using a non-disposable cloth towel, which may respond to cultural, socioeconomic or environmental awareness factors, and noted that this towel was not frequently replaced. Although the use of disposable paper tissues is commonly recommended over cloth towels, reusable single serve cloth towels may also be an option. Indeed, Patrick and co-workers noted a reduction in 99.8%, 94.0% and 99.0% in the level of bacterial translocation to samples of skin, food and utilities, respectively, when participants used a reusable single-serve cloth towel to dry their hands, as compared with touching the same samples with wet hands [38]. Similarly, other authors have observed that drying hands with single use paper tissues of good quality was able to reduce the number of potential pathogens remaining on the wet skin of the hands [19]. Moreover, the same tissue may be employed to open the storage case of the CLs and to manipulate the controls of the water tap, to reduce the chance or re-contamination after washing hands. The use of air dryers, reported by one respondent, is highly unadvisable, as they may become themselves contaminated and, therefore, aid in the dispersion of germs [39].

During the practical demonstration, less than one fourth of participants showed satisfactory hand washing habits and more than half of them displayed incorrect drying routines. Thus, even if these results were obtained from a small subset of the respondents of the initial survey, they evidence that poor hand hygiene is probably predominant. Previous researchers have noted that a practical demonstration is a more reliable indicator of hand washing performance than self-reported surveys, as many CL wearers do wash their hands but fail to do so effectively enough to remove pathogens [40]. Moreover, given the potential influence of the Hawthorne effect, that is, of participants displaying better performance than they would under normal conditions at home, because of being aware of the presence of the observer [41], it may be assumed that daily private performance is even less adequate. In effect, within a healthcare setting, previous researchers have noted that the sole presence of observers led to a 2.5-fold increase in good hand hygiene practices, even without additional interventions [42]. In another study conducted at a tertiary academic medical center, the proportion of correctly performed hand hygiene practices increased by 15% when auditors were present on the wards [43]. This study also revealed that the impact of the Hawthorne effect on hand hygiene was inversely related to the initial performance levels, with units with the lowest baseline hand hygiene rates showing the most significant improvements when auditors were present [43]. To minimize the Hawthorne effect, future research might benefit from employing a concealed video camera to record the CL care habits of participants during practical demonstrations.

In view of the present findings, a short list of recommendations regarding hand hygiene for daily and non-disposable soft CL users is provided in Table 2, adapted, in part, from the overall recommendations provided by McMonnies in 2012 and from the WHO guidelines published in 2009 [9,15].

### Strengths and Limitations

This study is not devoid of limitations. For instance, only the hand hygiene habits of daily and monthly replacement soft CL wearers were explored, without the distinction of the type of maintenance solution (multipurpose solution, hydrogen peroxide, etc.). It may be argued that, as other CL modalities, such as rigid corneal or scleral CLs, require different cleaning and maintenance procedures related to their longer replacement schedules, the implications for those patients of the poor hand hygiene procedures disclosed in this research are of higher clinical relevance. It must also be noted that the findings of the second part of the study must be interpreted with caution, as a selection bias of the participants attending the practical demonstration may not be ruled out. Indeed, although all respondents to the initial survey were invited to participate in the practical demonstration, actual proximity to the research lab may have been determinant to define this subset of participants. Moreover, the demographic and CL replacement characteristics of these participants were found to be different from those of the overall sample, and other factors, such as their willingness to share their contact details with the researchers, could have introduced confounding variables. In addition, given that participation was voluntary, CL wearers willing to participate may have been those more inclined to share their “proper” CL care habits with the investigators, although the real purpose of the practical demonstration was not divulged beforehand. 

Regarding sample size, although the required sample size was estimated at 385, the final sample of survey respondents was 203, with 198 valid surveys after revision. This sample size would result in a margin of error of approximately 7%, larger than the assumed 5% used for sample size estimation. However, it may be noted that this estimation was conducted considering the total number of CL wearers in Spain, that is, including other CL modalities such as rigid corneal CLs and orthokeratology, although wearers of these CLs modalities are a minority in Spain. As for the practical demonstration, sample calculation was not conducted; however, given the ubiquity of incorrect hand hygiene practices described in the literature, it was assumed that with a sample size of 20–30 participants, a clearer picture might emerge.

## 5. Conclusions

In conclusion, hand hygiene habits in a sample of Spanish soft CL wearers were found to be unsatisfactory, mainly regarding hand drying and hand washing prior to storage case manipulation. Given that poor compliance with hand hygiene may be considered a modifiable aspect of CL wear, practitioners and CL specialists are encouraged to pay particular attention to the instructions they provide their patients and to reinforce this information at every available opportunity through practical in-office demonstrations. 

## Figures and Tables

**Table 1:** Common hand hygiene practices in a sample of Spanish soft contact lens (CL) wearers.

Concept	Practice	n (%)
Hand washing and CLs (n = 198)	Always washed hands prior to inserting and removing CLs	141 (71.1%)
	Washed hands only prior to inserting CLs	21 (10.6%)
	Washed hands only prior to removing CLs	1 (0.5%)
	Washed hands only occasionally before handling CLs	32 (16.1%)
	Never washed hands before handling CLs	3 (1.7%)
Hand drying (n = 161)	Dried hands using a non-disposable cloth towel	132 (82.1%)
	Dried hands using disposable paper tissue	28 (17.3%)
	Dried hands with an air drier	1 (0.6%)
Hand washing and storage case (n = 156)	Always washed hands prior to handling storage case	70 (44.8%)
	Washed hands only occasionally before handling storage case	43 (27.6%)
	Never washed hands before handling storage case	43 (27.6%)

**Table 2:** Hand washing and drying tips for patients of soft daily and monthly disposable contact lenses (adapted from McMonnies, 2012, and the WHO guidelines, 2009 [9,15]).

Overall	1. Patients need to understand the reasons for good hand hygiene.
	2. Periodic reminders and practical demonstrations at all aftercare visits are recommended.
	3. Adequate language, positive message, reinforced with written instructions, should be employed.
Hand washing	1. Wash hands prior to any interaction with the eyes, contact lenses and/or accessories such solution bottles and storage cases.
	2. Start by rinsing hands to remove excess dirt.
	3. Always use sufficient soap and water.
	4. Rub the soap lather over all the surface of the hands, paying particular attention to fingernails.
	5. Use abundant running water and rubbing to rinse hands.
Hand drying	1. Dry hands with a disposable paper tissue or a small reusable single-serve cloth towel only used for CL-related purposes.
	2. Avoid non-disposable cloth towels and air driers.
	3. Use the same paper tissue to open the storage case, turn off the water tap and touch the door handle when exiting the bathroom (if applicable).
Other aspects	1. Fingernails should be kept short, and artificial cosmetic nails should be avoided.
	2. Avoid using alcohol-based hand rubs, as they may irritate eyes and damage contact lenses.

## Data Availability

Data are available from the authors upon reasonable request.
